# Revisiting the Karyotypes of Alligators and Caimans (Crocodylia, Alligatoridae) after a Half-Century Delay: Bridging the Gap in the Chromosomal Evolution of Reptiles

**DOI:** 10.3390/cells10061397

**Published:** 2021-06-05

**Authors:** Vanessa C. S. Oliveira, Marie Altmanová, Patrik F. Viana, Tariq Ezaz, Luiz A. C. Bertollo, Petr Ráb, Thomas Liehr, Ahmed Al-Rikabi, Eliana Feldberg, Terumi Hatanaka, Sebastian Scholz, Alexander Meurer, Marcelo de Bello Cioffi

**Affiliations:** 1 Laboratório de Citogenética de Peixes, Departamento de Genética e Evolução, Universidade Federal de São Carlos, São Carlos 13565-905, Brazil; vanessacristina.sales@gmail.com (V.C.S.O.); bertollo@ufscar.br (L.A.C.B.); hterumi@yahoo.com.br (T.H.); mbcioffi@ufscar.br (M.d.B.C.); 2 Department of Ecology, Faculty of Science, Charles University, 12844 Prague, Czech Republic; altmanova.m@gmail.com; 3 Laboratory of Fish Genetics, Institute of Animal Physiology and Genetics, Czech Academy of Sciences, 27721 Liběchov, Czech Republic; rab@iapg.cas.cz; 4 Laboratório de Genética Animal, Coordenação de Biodiversidade, Instituto Nacional de Pesquisas da Amazônia, Manaus 69083-000, Brazil; patrik.biologia@gmail.com (P.F.V.); feldberg@inpa.gov.br (E.F.); 5 Institute for Applied Ecology, Faculty of Science and Technology, University of Canberra, Bruce, ACT 2617, Australia; tariq.ezaz@canberra.edu.au; 6 Institute of Human Genetics, Jena University Hospital, Am Klinikum 1, 07747 Jena, Germany; ahmedgenetic@hotmail.com; 7 An der Nachtweide 16, 60433 Frankfurt, Germany; chinemys@web.de; 8 Alfred Nobel Strasse 1e, 55411 Bingen am Rhein, Germany; ameurer@online.de

**Keywords:** Alligatoridae, cytogenomics, chromosome, molecular cytogenetics

## Abstract

Although crocodilians have attracted enormous attention in other research fields, from the cytogenetic point of view, this group remains understudied. Here, we analyzed the karyotypes of eight species formally described from the Alligatoridae family using differential staining, fluorescence in situ hybridization with rDNA and repetitive motifs as a probe, whole chromosome painting (WCP), and comparative genome hybridization. All Caimaninae species have a diploid chromosome number (2n) 42 and karyotypes dominated by acrocentric chromosomes, in contrast to both species of Alligatorinae, which have 2n = 32 and karyotypes that are predominantly metacentric, suggesting fusion/fission rearrangements. Our WCP results supported this scenario by revealing the homeology of the largest metacentric pair present in both *Alligator* spp. with two smaller pairs of acrocentrics in Caimaninae species. The clusters of 18S rDNA were found on one chromosome pair in all species, except for *Paleosuchus* spp., which possessed three chromosome pairs bearing these sites. Similarly, comparative genomic hybridization demonstrated an advanced stage of sequence divergence among the caiman genomes, with *Paleosuchus* standing out as the most divergent. Thus, although Alligatoridae exhibited rather low species diversity and some level of karyotype stasis, their genomic content indicates that they are not as conserved as previously thought. These new data deepen the discussion of cytotaxonomy in this family.

## 1. Introduction

Unlike other vertebrates that underwent substantial diversification, extant crocodilian species have maintained morphological and ecological similarities for almost 100 million years (Myr) [1,2,3,4]. Crocodilians along with dinosaurs, pterosaurs, and birds form a monophyletic clade known as archosaurs. They represent a bridge between recent birds and non-avian recent reptiles; in support of this premise, the evidence from molecular phylogenetics indicates that crocodilians and birds form a monophyletic clade [5,6,7,8].

The order Crocodylia is a useful model for biogeographic studies, as its species show a circumtropical distribution, with at least one extant representative in each continent, except for Europe and Antarctica [9,10]. Such circum-oceanic distribution, combined with the ancient age and distinct phylogenetic position, make crocodilians an attractive model to understand evolutionary and biogeographic characteristics of ancient vertebrates, including the fact that they demonstrate the past dispersal events of many vertebrate lineages. Although their biogeography is still a mystery, some recent studies proposed that a relatively recent trans-Atlantic crossing, from Africa to the New World and from Indopacific to the New World likely occurred for certain crocodile species [4,11,12].

**Figure 1:**
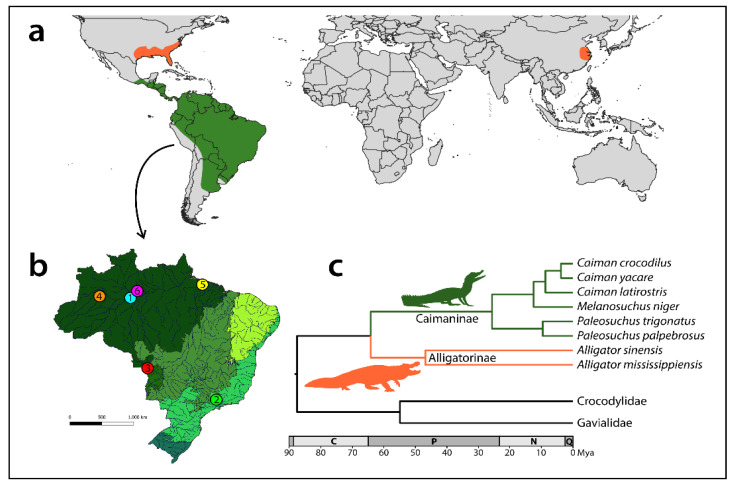
Recent distribution (**a**), sampling sites (**b**), and phylogenetic relationships (**c**) of Alligatoridae species. Map of Brazil highlighting the collection sites (colored circles) of the Caimaninae species analyzed in the present work, namely: 1. *Caiman crocodilus* (light blue circle); 2. *Caiman latirostris* (green circle); 3. *Caiman yacare* (red circle); 4. *Melanosuchus niger* (orange circle); 5. *Paleosuchus palpebrosus* (yellow circle); and 6. *Paleosuchus trigonatus* (pink circle). The map was created using QGis 3.4.3 and Adobe Photoshop CC 2020 software. Adapted time-calibrated phylogenetic tree for the order Crocodylia, focusing on Alligatoridae, based on data generated by [12] for an alternative dating see [8]. C—Cretaceous, P—Paleogene, N—Neogene, Q—Quaternary, and Myr—million years ago.

Crocodylia is divided into three families (Figure 1): Crocodylidae, Gavialidae, and Alligatoridae, and the number of currently described species ranges from 23 to 26 [2,13,14,15,16,17,18,19,20]. Crocodylidae is represented by three genera, namely *Crocodylus*, *Mecistops,* and *Osteolaemus*, and it is composed of 16 species [14,21]—but potentially reaching 17 depending on formal taxonomic review and species validation [15,16,21,22]. This family is distributed in Asia, Australia, Africa, and America [11]. Gavialidae has only one species, the Indian gharial *Gavialis gangeticus*, which is native to India and Nepal [23,24]. However, the phylogenetic position of the false gharial *Tomistoma schlegelii*, a species widespread in South Asia, remains under debate, although molecular analyses put *Tomistoma* into Gavialidae [8,12]. The third family, Alligatoridae, consists of eight species distributed in four genera: *Alligator,* forming the monogeneric subfamily Alligatorinae, and *Melanosuchus*, *Paleosuchus*, and *Caiman,* belonging to Caimaninae [8,20] (but see [25,26] suggesting the existence of other cryptic species). Except for *Alligator*, where *A. mississippiensis* and *A. sinensis* are restricted to the Southeastern United States and China, respectively, the distribution of Caimaninae species ranges from Mexico to South America, being especially widespread in Brazil [2,12]. Yet, e.g., *Caiman crocodilus* has been introduced also to other regions such as Puerto Rico, Cuba, and Florida [27]. *A. sinensis* represents one of the most endangered crocodilian species, listed as a CITES Appendix I species, in the category ‘critically endangered’ by the International Union for Conservation of Nature (IUCN). The most recent survey performed in 2015 indicated that its wild population was estimated at 136–173 individuals (32 adults) concentrated in a small region in southeastern Anhui Province [28]—a fraction of its former distribution [29].

Reptiles show a vast diversity in diploid chromosome number (2n) and karyotype morphology, with various combinations of macro- and microchromosomes, as well as sex determination systems [30,31,32]. Molecular cytogenetic techniques have been largely applied, providing better insight into their chromosomal evolution (reviewed in [31]). In addition to the mapping of repetitive DNA sequences and whole chromosome painting (WCP) experiments, the recent use of the comparative genomic hybridization (CGH) has allowed us to compare the degree of genome similarity at the level of repetitive DNA content among phylogenetically related species [33,34,35,36,37,38,39,40,41,42]. However, cytogenetic data for Crocodylia are usually restricted to the description of the 2n, karyotype composition, and some conventional banding [43,44,45,46]; only few studies used molecular cytogenetic tools [45,47,48,49,50,51].

The 2n of crocodilians ranges from 30 to 42 and positively correlates with the ratio of acrocentrics in the complement. Together with low variability in the number of chromosome arms, NF = 56–60/62 (NF, *nombre fondamental*), it suggests that the karyotypes evolved mostly by fusion/fission [17,44,52]. Their karyotype structure, however, lacks typical reptile dot-shaped microchromosomes. Such pattern strikingly contrasts with the one found in birds and turtles, once their karyotypes comprise, with few exceptions, at least 50 chromosomes including a small number of macrochromosomes and many indistinguishable microchromosomes [31,40,52,53]. 

In addition, the G-banding pattern is very conservative in Crocodylia, showing similar pattern among chromosomes of *Crocodylus porosus*, *Crocodylus johnstoni,* and *Caiman crocodilus* [45], pointing to a generally conserved chromosome/replication structure without apparent inter- or intra-chromosomal reshuffling among species. Another notable crocodilian feature is that the whole order likely shares the environmental sex determination (ESD). The effect of incubation temperature on the offspring sex ratio was observed in several species and on the other hand, the sex chromosomes have not been identified in any of cytogenetically studied species [35,44,54,55,56].

Thus, based mostly on conventional methods, the cytogenetics of Crocodylia still represents a kind of missing piece in understanding of chromosomal evolutionary patterns in reptiles. To fill this gap, we analyzed the karyotype organization of all taxonomically recognized Alligatoridae species by differential conventional stainings and up to date molecular cytogenetic techniques, namely the chromosomal mapping of repetitive DNA sequences, WCP, and CGH methods. The results are compared and discussed with previously published data. This study is part of a series on cytogenetics and cytogenomics of crocodilians.

## 2. Materials and Methods

### 2.1. Sampling Species, Mitotic Chromosome Preparations, C-Banding, and CMA3 Staining

Blood samples were obtained from free-living South American animals with the authorization of the environmental agency ICMBIO/SISBIO (License nº 71857-7) and SISGEN (ABFF266). Blood samples from *Alligator sinensis* came from the animals legally kept in Europe (CITES certificate number EU 0228-1057/14, ES-CC-00041/07C, ES-CC-00036/07C, 50721-18, DE-DA190814-5, DE-DA190814-6). The collection sites, number, and sex of individuals are summarized in Figure 1 and Table 1. All experiments followed ethical conduct and were approved by the Ethics Committee on Animal Experimentation of the Universidade Federal de São Carlos (Process number CEUA 4617090919). No animals were seriously harmed, and all free-living individuals were released back to their respective collection sites.

Chromosomal preparations were obtained by means of in vitro blood cultures [57,58]. Constitutive heterochromatin was identified by the C-banding method according to [59], CG- and AT-rich chromosomal regions were highlighted using Chromomycin A_3_ (CMA3) and 6-diamidino-2-phenylindole (DAPI), respectively [60].

### 2.2. FISH with rDNA and Repetitive Motifs

We isolated 18S rDNA and (TTAGGG)_n_ probes from the genome of *C. latirostris* according to [61] and [62], respectively, and directly labeled with Atto550-dUTP using the Nick-Translation mix kit (Jena Bioscience, Jena, Germany), following manufacturer’s instructions. The repetitive motif (CGG)_10_ was directly labeled with Cy3 during the commercial synthesis, as described by [63]. FISH followed the protocol described by [64], with minor modifications according to [41]. Chromosomes were counterstained with DAPI (1.2 µg/mL), and the slides were mounted in an antifade solution (Vector, Burlingame, CA, USA).

### 2.3. Microdissection and Whole Chromosome Painting

We selected chromosome No. 1 of *A. mississippiensis* for experiments of cross-species painting (sometimes also referred as Zoo-FISH), as it could be unambiguously identified as the largest chromosome of each metaphase. Twelve copies of this chromosome were isolated by glass-needle based microdissection and amplified using the procedure described in [65]. The probe was referred to as AMI-1, and it was labeled with Spectrum Green-dUTP (Vysis, Downers Grove, IL, USA) in a secondary Degenerate Oligonucleotide-Primed Polymerase Chain Reaction (DOP PCR) using 1 µL of the primarily amplified product as template DNA [65]. Zoo-FISH with the AMI-1 probe was applied on chromosomal preparations from all Alligatoridae species as described in [64].

### 2.4. Comparative Genomic Hybridization

We focused on interspecific genomic comparisons by CGH. The female-derived total genomic DNA (gDNA) of one representative of each genus (namely: *C. latirostris, P. palpebrosus*, *M. niger,* and *A. sinensis*) was compared with the female-derived gDNA of *C. yacare*. For that, separate CGH experiments were performed against metaphase chromosomes of *C. yacare*. The gDNAs were extracted from blood using the standard phenol-chloroform-isoamyl alcohol method [66]. The gDNA of *C. yacare* was labeled with Atto550-dUTP by means of nick translation (Jena Bioscience, Jena, Germany), while the gDNAs of the other species were labeled with Atto488-dUTP also by nick translation (Jena Bioscience, Jena, Germany). 

In all experiments, repetitive DNA sequences were blocked by unlabeled C0t-1 DNA (i.e., fraction of genomic DNA enriched for highly and moderately repetitive sequences), prepared according to [67]. The final probe cocktail for each slide was composed of 500 ng of gDNA of *C. yacare* + 500 ng of gDNA corresponding to one of the comparative species + 6 μg of female-derived C0t-1 DNA of each species. The probe was ethanol-precipitated, and the dry pellets were resuspended in hybridization buffer containing 50% formamide, 2× saline-sodium citrate buffer, 10% sodium dodecyl sulphate, 10% dextran sulfate and Denhardt’s buffer, pH 7.0. The chosen ratio of probe vs. C0t-1 DNA amount was based on the previous experiments performed in reptiles [41]. The CGH experiments followed the methodology described in [68].

### 2.5. Microscopic Analyses and Image Processing

At least 10 metaphase spreads per individual were analyzed to confirm the 2n chromosome number, karyotype structure, and FISH results. Images were captured using an Olympus BX50 microscope (Olympus Corporation, Ishikawa, Japan) with a CoolSNAP CCD camera (Teledyne Photometrics, Tucson, AZ, USA), and the images were processed using Image Pro Plus 4.1 software (Media Cybernetics, Silver Spring, MD, USA). The chromosomes were classified as metacentric (m), submetacentric (sm), and acrocentric (a) according to [69].

## 3. Results

### 3.1. Karyotypes, C-Banding, and Chromomycin A_3_-Staining

The 2n for all Caimaninae species equaled 42 for both sexes. Their karyotypes were composed of 24a + 18m/sm chromosomes in *C. crocodilus* and *C. latirostris*; 28a + 14m/sm chromosomes in *C. yacare*, *P*. *palpebrosus,* and *P*. *trigonatus*, and 32a + 10m/sm chromosomes in *M. niger*. North American *A. mississippiensis* and Chinese *A. sinensis* had 2n = 32 and their karyotypes were invariably formed by 4a + 28m/sm chromosomes. No intraindividual chromosomal variability was observed, neither that between males nor females, indicating the absence of heteromorphic sex chromosomes. 

**Figure 2:**
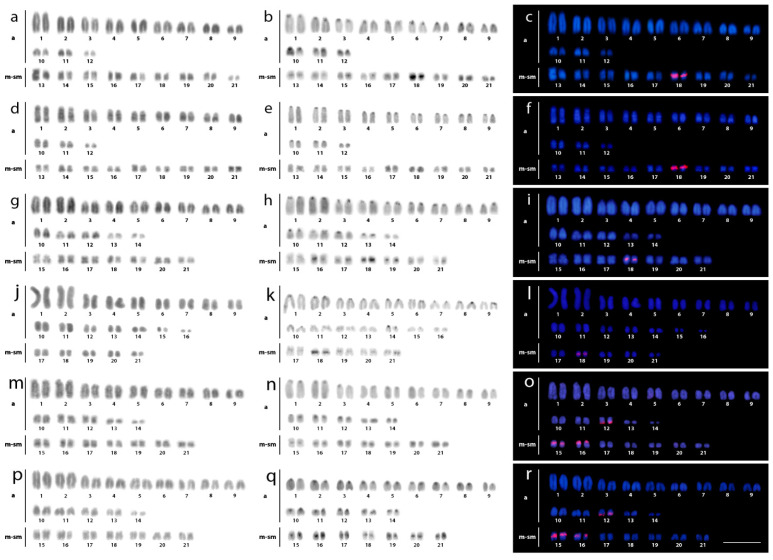
Female karyotypes of *Caiman crocodilus* (**a**–**c**), *C. latirostris* (**d**–**f**), *C. yacare* (**g**–**i**), *Melanosuchus niger* (**j**–**l**), *Paleosuchus palpebrosus* (**m**–**o**) and *P. trigonatus* (**p**–**r**) arranged after inverted-DAPI staining (**a**,**d**,**g**,**j**,**m**,**p**), C-banding (**b**,**e**,**h**,**k**,**n**,**q**) and FISH with 18S rDNA (red) probe (**c**,**f**,**i**,**l**,**o**,**r**). Bar = 20 μm.

**Figure 3:**
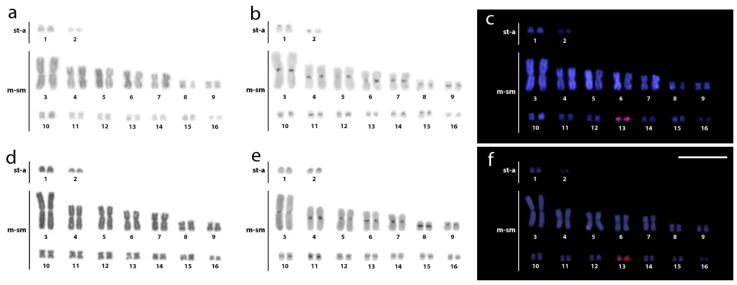
Female karyotypes of *Alligator mississippiensis* (**a**–**c**) and *A. sinensis* (**d**–**f**) arranged after inverted-DAPI staining (**a**,**d**), C-banding (**b**,**e**), and FISH analysis with 18S rDNA (red) probe (**c**,**f**). Bar = 20 μm.

**Figure 4:**
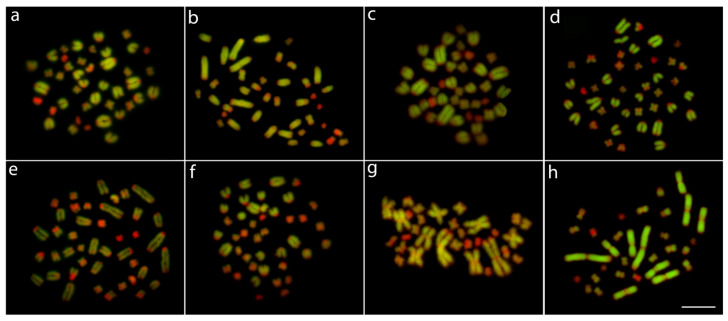
Metaphase chromosomes from females of *Caiman crocodilus* (**a**), *C. latirostris* (**b**), *C. yacare* (**c**), *Melanosuchus niger* (**d**), *Paleosuchus palpebrosus* (**e**), *P. trigonatus* (**f**), *Alligator mississippiensis* (**g**), and *A. sinensis* (**h**) after CMA3/DAPI banding (GC- and AT-rich regions pseudocolored in red and green, respectively). Bar = 20 μm.

Therefore, these data extended and consolidated previous information for these species [43,44,45,46,51,54,70,71,72,73,74]. C-positive heterochromatic bands were detected at the centromeric regions of almost all chromosomes, with more prominent bands in the rDNA-bearing chromosome pair(s) (Figure 2 and Figure 3). CMA3+ bands occurred in almost all centromeric regions, with very bright signals observed in the smallest chromosomes (Figure 4).

### 3.2. Fluorescence In Situ Hybridization (FISH) Mapping of Repetitive DNAs

Two distinct patterns of distribution of 18S rDNA sites were observed. In all three *Caiman* spp., *M. niger,* and both *Alligator* spp., these sites were located in the centromeric region of a single metacentric pair No. 18. However, in karyotypes of both *Paleosuchus* spp., six chromosomes displayed 18S rDNA sites (Figure 2 and Figure 3). (CGG)_10_ showed positive hybridization signals in all species, ranging from four to eight chromosomes containing these motifs. 

**Figure 5:**
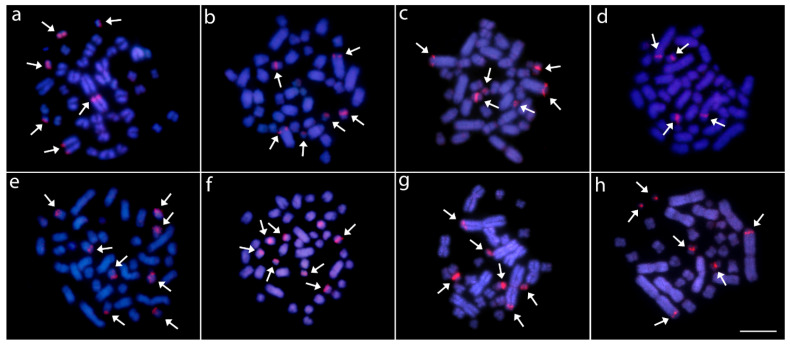
Metaphase chromosomes from females *Caiman crocodilus* (**a**), *C. latirostris* (**b**), *C. yacare* (**c**), *Melanosuchus niger* (**d**), *Paleosuchus palpebrosus* (**e**), *P. trigonatus* (**f**), *Alligator mississippiensis* (**g**), and *A. sinensis* (**h**) hybridized with (CGG)_n_ microsatellite probe (red). Chromosomes were counterstained with DAPI (blue). Arrows indicate the CGG+ signals. Bar = 20 μm.

In addition to signals on four small chromosomes (only two in *Melanosuchus*), all the studied species shared a signal in the terminal position of the largest chromosome pair, except for both *Paleosuchus* spp. where the signal with these particular sites was lacking, but with signals present on four other small chromosomes instead (Figure 5). FISH with the telomeric (TTAGGG)_n_ probe performed in four species (*C. latirostris*, *P. palpebrosus*, *A. mississippiensis,* and *A. sinensis*) hybridized only to the telomeric regions of all chromosomes, without interstitial telomeric sites (ITSs) (Figure S1).

### 3.3. WCP of AMI-1 Probe

**Figure 6:**
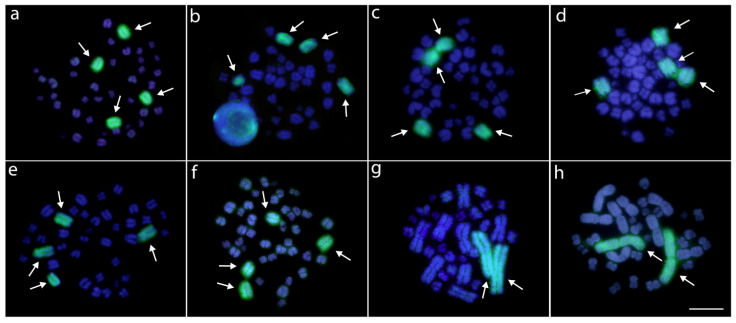
Zoo-FISH experiments with the AMI-1 painting probe (green) applied against female metaphase plates of *Caiman crocodilus* (**a**), *C. latirostris* (**b**), *C. yacare* (**c**), *Melanosuchus niger* (**d**), *Paleosuchus palpebrosus* (**e**), *P. trigonatus* (**f**), *Alligator mississippiensis* (**g**), and *A. sinensis* (**h**). Chromosomes were counterstained with DAPI (blue). Arrows indicate the chromosomes painted with AMI-1 probe. Bar = 20 μm.

The AMI-1 probe, when applied against metaphase chromosomes of *A. mississippiensis* and *A. sinensis*, completely painted the largest metacentric chromosome pair. Otherwise, its hybridization to Caimaninae species consistently painted two acrocentric chromosomal pairs (Figure 6).

### 3.4. Comparative Genomic Hybridization (CGH)

**Figure 7:**
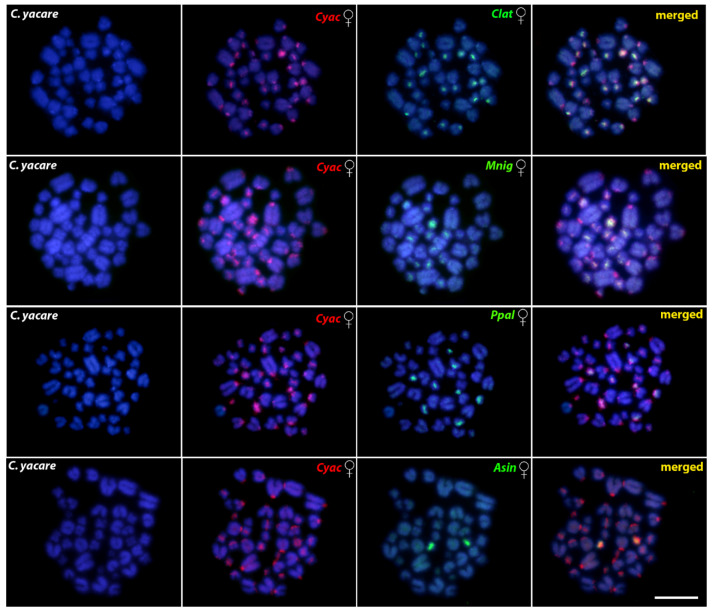
Mitotic chromosome spreads of *Caiman yacare* females after CGH—inter-specific hybridizations. First column: DAPI images (blue). Second column: hybridization pattern of the female-derived probe (red) of *C. yacare*. Third column: hybridization pattern of the female-derived probe (green) of each analyzed species. Fourth column: merged images of both genomic probes and DAPI staining. The common genomic regions are depicted in yellow. Clat = *C. latirostris,* Cyac = *C. yacare,* Mnig = *M. niger,* Ppal = *P. palpebrosus,* and Asin = *A. sinensis*. Bar = 10 µm.

The gDNA of *C. yacare* hybridized against its own chromosome complement highlighted abundant heterochromatic blocks in the centromeric regions of several chromosomes. The CGH experiments using gDNA probes from *C. latirostris* and *M. niger* on *C. yacare* chromosomes demonstrated several overlapping signals in the centromeric regions, indicating that the centromeres of these species are enriched in similar repetitive sequences, although *M. niger* in smaller amounts and/or in different repeats. However, *P. palpebrosus* and *A. sinensis* shared a little of repetitive content with *C. yacare*, restricted almost exclusively to the 18S rDNA-bearing pair(s), indicating their high degree of centromeric sequence differentiation (Figure 7).

## 4. Discussion

The evolutionary history of non-avian reptiles (turtles, crocodilians, and squamates) led to an accentuated asymmetric species-richness among its groups - from the 11.341 living species, only approximately 26 (0.23%) are crocodilians [20]. As the crocodylomorph group has inhabited the Earth for more than ~100 million years (Myr) and represents adaptations to different food and habitat niches, the intriguing open question is, why are there so few extant living crocodilian species? [4]. Alfaro et al. [75] found that they are diversifying 1000-times slower than expected. This goes also together with a low disparity rate, being about 10,000 times smaller than that of other groups having the same evolutionary time scale, such as birds and lepidosaurs [76,77].

This low rate of diversification over time may somehow be related to the crocodilians karyotype evolution [1,44]. While birds (2n = 40–142), turtles (2n = 28–68), and squamate reptiles (2n = 16–62) have higher levels of variability in diploid chromosome number and karyotype morphology, crocodilians present much less variation in their karyotypes [30,31,52,53,78,79]. In fact, the cytogenetic investigation of 23 out of 26 crocodilian species reveals general karyotypic patterns as they present a lower 2n = 30–42 and the predominance of a few large chromosomes, together with the absence of dot-shaped microchromosomes in their karyotypes [17]. The predicted ancestral karyotype for archosaurs + turtles shows at least eight pairs of macrochromosomes and many indistinguishable microchromosomes. In this way, the crocodilian lineage strikingly diverges from that ancestral pattern, since all microchromosomes disappeared by fusion events among them [49].

While 2n ranges from 30 to 34 in representatives of Crocodylidae and Gavialidae (except for the African dwarf crocodile *Osteolaemus tetraspis*, with 2n = 38), two major pathways can be recognized in the chromosomal differentiation inside Alligatoridae: (i) conservation of the low diploid number (2n = 32) and karyotypes composed of mostly bi-armed chromosomes in *A. mississippiensis* and *A. sinensis* ([44], this study); and (ii) a higher chromosome number 2n = 42 in *Caiman*, *Melanosuchus,* and *Paleosuchus* spp., with karyotypes dominated by acrocentric elements ([44,51], this study). 

This suggests that extensive chromosomal rearrangements must have occurred in the karyotype evolution at the split of Alligatorinae and Caimaninae. Since a lower 2n can be recognized as the ancestral condition for the Crocodylia order [17], it is likely that karyotype diversification in the common ancestor of all Caimaninae species was accompanied by a series of fission events after the split of *Alligator* (~70 Myr) [80], thus, increasing the 2n value. Our Zoo-FISH results support this scenario by highlighting the homeology of the largest metacentric chromosomal pair present in both *Alligator* species with four smaller acrocentric chromosomes in all three Caimaninae genera (Figure 6). 

The alternative hypothesis considering 2n = 42 as the plesiomorphic state might be valid under this scenario with caimans being a sister lineage to all other crocodilians. However, regarding current phylogeny, the Alligatorinae + Caimaninae is sister to Crocodylidae + Gavialidae, the alternative hypothesis is supported neither by 2n = 30–32 widely distributed in all other Crocodylia lineages, nor by our telomeric FISH results. Interstitial telomeric sequences (ITS) might reflect the remnants of telomeres in neo-chromosomes originated by the chromosome fusion of two ancestral chromosomes [81]. However, in alligators, only the standard terminal topology of telomeres is found in all chromosomes, with no ITS indicating probable fusion points in the large metacentrics (Figure S1). Therefore, although crocodilians do not exhibit a high karyotype divergence among their living species, the various chromosomal rearrangements, especially those present in the caimans, might have played a role in the species radiation. In this context, it cannot be ruled out that the increase in 2n may have favored a higher rate of recombination, due to an increase in independent chromosomal segregation during meiosis, thus, likely bringing some advantage to the colonizing specimens of the new South American environment.

In most reptiles and birds, rDNA clusters are frequently located in a single chromosome pair [53,82,83], with few exceptions showing an amplified number of rDNA sites in snakes [84,85], lizards [86], turtles [87], and birds (reviewed in [53]). Here, this same general pattern is also followed by all Alligatoridae species, except both examined *Paleosuchus* species, in which three chromosome pairs bear these sequences (Figure 2). In fact, single rDNA sites are usually found in old lineages, as also observed in ancient fish groups, e.g., ancient non-teleost actinopterygian fishes [83].

Chromosomes displaying sequences shorter than 30 Mb—the microchromosomes—are widely documented in almost all vertebrate groups [88]. Specifically, the extant sauropsids (some reptiles and birds) generally also present these major components in their karyotypes [89,90]. In turtles and birds, microchromosomes exhibit a higher gene density and GC-content [91,92,93,94,95]. Similarly, microchromosomes of the squamate reptiles also display GC-rich content in comparison with macrochromosomes, although their CMA3+ signals are never as bright as in birds (e.g., [58]), a difference in the chromosome/genome composition that is also confirmed by genomic approaches [88]. 

Our results demonstrated that all Alligatoridae species also possess a high accumulation of GC-positive blocks on small chromosomes with strong and sharp signals co-localized with the 18S rDNA clusters. This pattern can be ascribed as unique, showing a similar and preserved G-banding pattern in the whole order [45]. The poorer GC-positive pattern in the largest chromosomes indicates sequence-specific regions, a characteristic that influences the reduction of the chromosomal recombination rate [96].

Turtles and crocodiles have larger genomes, with a more variable DNA content when compared to other reptiles, mainly due to the tendency to accumulate and preserve repetitive DNA [30,73]. They also have few Simple Short Repeats (SSR) when compared to other lizard species [97]. In fact, microsatellites or SSRs are one of the broadest and most representative DNA class in several vertebrate species [98,99,100] and are known to display a dynamic role in the genomic functioning [101]. Due to their high rates of mutation and fast evolution, usually modulated by an association with mobile elements [102,103,104,105], the genome landscapes may vary drastically among related groups. 

Non-avian reptiles, for instance, stand out in such a scenario [99,106,107]. Interestingly, crocodilians seem to be the poorest group concerning SSR content among avian and non-avian reptiles [7,99], being that the density and content of SSR repeats are relatively similar among species, with slow rates of mutation and evolution when compared to other lineages. Alligators and caimans constitute a unique group with any SSR mapping data up to now, and (CGG)_n_ showed some accumulation but in only two chromosome pairs in *M. niger*, three pairs in *Caiman* and *Alligator* species, and four pairs in *Paleosuchus* species.

Although conservative, the repetitive DNA fraction shows divergences among Caimaninae species, as demonstrated by our CGH experiments where both *Paleosuchus* species stand out as the most divergent ones (Figure 7). Following the phylogenetic hypothesis, the cytogenetic similarity of centromeric repeats between *Caiman* and *M. niger* (diverged at ~12 Myr) was higher compared to *Paleosuchus*, which are phylogenetically distant and diverged from the genera *Caiman* + *Melanosuchus* at ~22 Myr [12]. The extensively shared cytogenetic features of *Caiman* and *Melanosuchus* are also supported by the CGH experiments, with little differences in the overall hybridization patterns demonstrating a high degree of sequence homology. 

Although the evolutionary divergence time among the Caimaninae may not have been long enough for the fixation of karyotype differences regarding the 2n and karyotype composition, it allowed the changes in the patterns of the repetitive DNA fraction due to the different evolutionary dynamics. The CGH experiments between Caimaninae and Alligatorinae suggested an advanced stage of sequence divergence, except for the bright signals, highly likely corresponding to NOR sites (as might be compared with previous rDNA FISH analysis). Such a similar scenario was already previously reported for distantly related or substantially diverged genomes [108,109,110].

The progressive temporal reduction of the chromosome homology in Caimaninae appears to have occurred slowly and suggests a tendency of an internal reorganization in chromosomes operating to gradually reduce the degree of collinearity and conserved synteny, as observed in several animal groups [111]. Karyotype stasis, characterized by the absence of conspicuous modifications throughout the evolutionary history, has already been discovered in many biological groups, such as plants [112,113,114], amphibians [115,116], birds [117], and fishes [118,119]. Among fishes, for example, the Gondwanan notopterids (Teleostei, Osteoglossiformes), whose species diverged by more than 100 Myr, display conserved karyotypes along such evolutionary time scales, with only slight disturbances of collinearity [110,120]. 

However, the maintenance of such conservative traits for millions of years is not always well understood and suggests some possible causes, such as stabilizing selection [121], a punctuated model of evolution [122], or orthoselective processes [123,124]. In crocodilians, the ancient periods of evolutionary divergence among its lineages do not support the hypothesis that karyotype stasis is a byproduct of recent processes of speciation. As karyotype and chromosome diversifications may accompany speciation [125,126,127], the low crocodilian species diversity might be directly linked with their karyotype features, which must have been influenced by the climactic fluctuations that occurred during the Cenozoic period [4].

## 5. Conclusions

This study is the first to offer reliable chromosomal data for all taxonomically recognized Alligatoridae species based on both conventional and molecular cytogenetic data and to provide a first view on the evolutionary history and chromosomal evolution of alligators and caimans. We observed a stable dichotomy among the genera *Alligator* (2n = 32) and *Caiman*, *Melanosuchus,* and *Paleosuchus* (2n = 42), where 2n = 32 represents the likely ancestral state, which is also supported by other chromosomal data. Therefore, karyotype diversification in Caimaninae was followed by a series of Robertsonian rearrangements in which centric fissions played a key role. Additional investigations into the relationships between North American and Chinese alligators with caimans in South America may provide further information on the role of biogeographic factors in karyotype differentiations in Crocodylia.

## Figures and Tables

**Table 1:** Species, sample size (N), sex and locality of the analyzed individuals.

Species	N	Locality/Origin of Samples	
① *Caiman crocodilus* (Spectacled caiman)	2♀, 2♂	Amazonas (BR) (Amazon Basin)	3°22′34.7″ S 60°19′20.7″ W
② *Caiman latirostris* (Broad-snouted caiman)	4♀, 6♂	São Paulo (BR) (Cerrado)	22°33′53.1″ S 48°00′35.2″ W
③ *Caiman yacare* (Yacare caiman)	2♀, 8♂	Mato Grosso (BR) (Pantanal)	16°19′32.0″ S 57°46′35.7″ W
④ *Melanosuchus niger* (Black caiman)	2♀, 2♂	Amazonas (BR) (Amazon Basin)	3°25′50.4″ S 66°02′35.0″ W
⑤ *Paleosuchus palpebrosus* (Cuvier’s dwarf caiman)	3♀, 3♂	Pará (BR) (Amazon Basin)	1°18′19.7″ S 48°19′05.0″ W
⑥ *Paleosuchus trigonatus* (Schneider’s smooth-fronted caiman)	3♀, 4♂	Amazonas (BR) (Amazon Basin)	3°06′52.0″ S 60°01′58.0″ W
*Alligator mississippiensis* (American alligator)	2♀, 2♂	Canberra University collection (Australia)	
*Alligator sinensis* (Chinese alligator)	4♀, 1♂, 1 unsexed	Private collections (Germany)	

## Data Availability

Not applicable.

## Supplementary Materials

The following are available online at https://www.mdpi.com/article/10.3390/cells10061397/s1, Figure S1: Metaphase plates of females from *C. latirostris* (**a**), *P. palpebrosus* (**b**), *A. mississippiensis* (**c**), and *A. sinensis* (**d**) with telomeric (TTAGGG)n probe (red). Bar = 5 µm.
