# Supplementary figures and images for: Revisiting the Karyotypes of Alligators and Caimans (Crocodylia, Alligatoridae) after a Half-Century Delay: Bridging the Gap in the Chromosomal Evolution of Reptiles
Source: Cells. 2021 Jun 5;10(6):1397. doi: 10.3390/cells10061397 (PMC8228166; doi:10.3390/cells10061397)

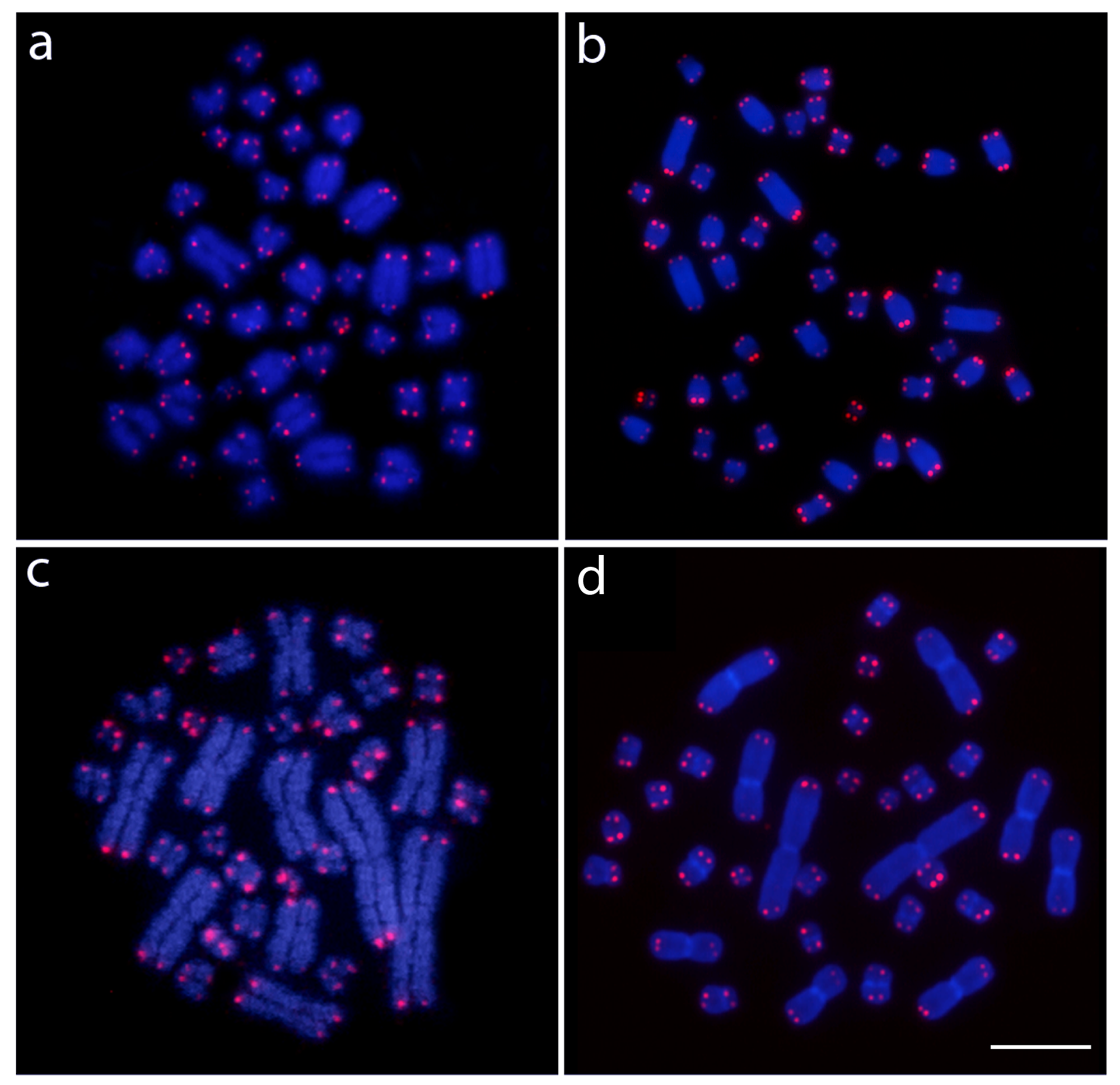

Supplement: Supplementary file 1 [file cells-10-01397-s001.zip › cells-1236511-supplementary.tif]
